# Association of premature ventricular complexes and risk of ischemic stroke: A systematic review and meta‐analysis

**DOI:** 10.1002/clc.23531

**Published:** 2020-12-16

**Authors:** Pongprueth Rujirachun, Phuuwadith Wattanachayakul, Prawut Phichitnitikorn, Nipith Charoenngam, Jakrin Kewcharoen, Arjbordin Winijkul

**Affiliations:** ^1^ Department of Microbiology, Faculty of Medicine, Siriraj Hospital Mahidol University Bangkok Thailand; ^2^ Department of Medicine, Faculty of Medicine, Siriraj Hospital Mahidol University Bangkok Thailand; ^3^ University of Hawaii Internal Medicine Residency Program Honolulu Hawaii USA; ^4^ Division of Cardiology, Department of Medicine, Faculty of Medicine, Siriraj Hospital Mahidol University Bangkok Thailand

**Keywords:** ischemic stroke, meta‐analysis, premature ventricular complex, risk factor

## Abstract

Recent studies have suggested that patients with premature ventricular complexes (PVCs) may have a higher risk of ischemic stroke. However, the data are limited and inconclusive. We conducted a systematic review and meta‐analysis to investigate the association between PVCs and the risk of ischemic stroke. A comprehensive literature review was conducted by searching for published articles indexed in MEDLINE and EMBASE databases from inception through September 25, 2020, to identify studies that compared the risk of ischemic stroke between patients with PVCs and individuals without PVCs. Pooled risk ratio (RR) and 95% confidence interval (CI) were calculated using a random‐effect, generic inverse variance method of Dersimonian and Laird. A total of four observational studies (2 prospective and 2 retrospective cohort studies) with 42 677 participants met the eligibility criteria and were included in the meta‐analysis. We found that patients with PVCs have a significantly higher risk of ischemic stroke than individuals without PVCs with the pooled RR of 1.31 (95% CI, 1.07–1.60, I^2^ = 43%). From our systematic review and meta‐analysis, we found that PVCs are associated with a higher risk of ischemic stroke. Whether this association is causal and how it should be addressed in clinical practice require further investigations.

## 1 INTRODUCTION

Premature ventricular complexes (PVCs) are a premature depolarization originating from ventricles and is the most common asymptomatic irregular ventricular activity on electrocardiogram (ECG).^1^, ^2^ PVCs are a well‐known predictor of cardiovascular morbidities and mortality, especially in patients with pre‐existing heart disease. From previous studies, the presence of high burden PVCs is associated with an increased risk of major cardiac events such as malignant dysrhythmia, cardiomyopathy, fatal coronary heart disease, and sudden cardiac death.^3^, ^4^, ^5^, ^6^, ^7^, ^8^ Nonetheless, the role of PVCs in the development of the cerebrovascular disease is less well studied.

Stroke is the leading cause of death and disability worldwide.^9^, ^10^ Approximately 90% of strokes are ischemic. However, the etiology of ischemic stroke remains unknown in 15% to 40%.^11^, ^12^ There are several verified risk factors as a target for ischemic stroke prevention, such as hypertension, diabetes mellitus, smoking, hypercoagulable states, and atrial fibrillation (AF).^13^, ^14^ Besides AF, other arrhythmias are less recognized as risk factors of ischemic stroke.

Interestingly, recent literature has suggested that PVCs may also be a risk factor for ischemic stroke, similarly to AF.^7^, ^15^, ^16^, ^17^ Nonetheless, the existing evidence is relatively limited and inconclusive. The current systematic review and meta‐analysis was conducted with the aim to comprehensively evaluate the risk of ischemic stroke among patients with PVCs compared to individuals without PVCs by identifying all relevant studies and combining their results.

## 2 METHODS

### 2.1 Literature search strategy

Three investigators (Pongprueth Rujirachun, Phuuwadith Wattanachayakul, and Prawut Phichitnitikorn) independently searched for published articles indexed in MEDLINE and EMBASE database from inception to September 25, 2020, using the search strategy that included the terms for PVCs and ischemic stroke. The search strategy is available as supplementary data 1. References of the included studies were also manually reviewed for additional eligible studies. This study was undertaken under the Preferred Reporting Items for Systematic Reviews and Meta‐Analyses (PRISMA) statement, which is available as supplementary data 2.

### 2.2 Selection criteria

To be eligible for the meta‐analysis, the study must be cohort study that investigated the association between PVCs and the risk of ischemic stroke. Eligible cohort study must consist of patients with PVCs and comparators without PVCs. Then, the eligible study must follow them until the occurrence of ischemic stroke or the end of the study. The eligible study must also provide the magnitude of association, which could be relative risk (RR), hazard ratio (HR), incidence rate ratio (IRR), or standardized incidence ratio (SIR) along with its corresponding confidence interval (CI).

All retrieved articles were reviewed independently by the first three investigators (Pongprueth Rujirachun, Phuuwadith Wattanachayakul, and Prawut Phichitnitikorn) for their eligibility. The last three investigators (Nipith Charoenngam, Jakrin Kewcharoen, and Arjbordin Winijkul) reviewed all the included studied again to ensure that the inclusion criteria were met and also served as the deciding votes when different determinations of study eligibility were made by the first three investigators. Newcastle–Ottawa quality assessment scale was used to assess the quality of the included cohort studies.^18^ This scale evaluates the quality of the included studies in three areas including recruitment of participants, comparability between the groups, and ascertainment of the outcome of interest for cohort study.

### 2.3 Data extraction

A standardized data collection form was used to extract the following information: last name of the first author, study design, year(s) of study, country of origin, year of publication, sample size, baseline characteristics of participants, methods used to identify and verify the diagnosis of PVCs and ischemic stroke, confounders that were adjusted and adjusted effect estimates with 95% CI. This data extraction was independently performed by the same three investigators (Pongprueth Rujirachun, Phuuwadith Wattanachayakul, and Prawut Phichitnitikorn) to minimize error. Any discrepancies found in the case record forms were resolved by referring back to the original articles.

### 2.4 Statistical analysis

Review Manager 5.3 software from the Cochrane Collaboration was used for data analysis. Point estimates and standard errors were extracted from individual studies and were combined using the generic inverse variance method as described by DerSimonian and Laird.^19^ Random‐effect model, rather than a fixed‐effect model, was used because the included studies were of different methodologies and background populations. HR of cohort study was used as an estimate for RR to calculate the pooled RR along with RR of cohort studies. Statistical heterogeneity was assessed using the Cochran's Q test. This statistic is complemented with the I^2^ statistic which quantifies the proportion of the total variation across studies that is due to heterogeneity rather than chance. A value of I^2^ of 0% to 25% represents insignificant heterogeneity, 26% to 50% low heterogeneity, 51% to 75% moderate heterogeneity, and > 75% high heterogeneity.^20^ The presence of publication bias would be assessed by visualization of the funnel plot if enough studies were eligible for the meta‐analysis.

## 3 RESULTS

**FIGURE 1:**
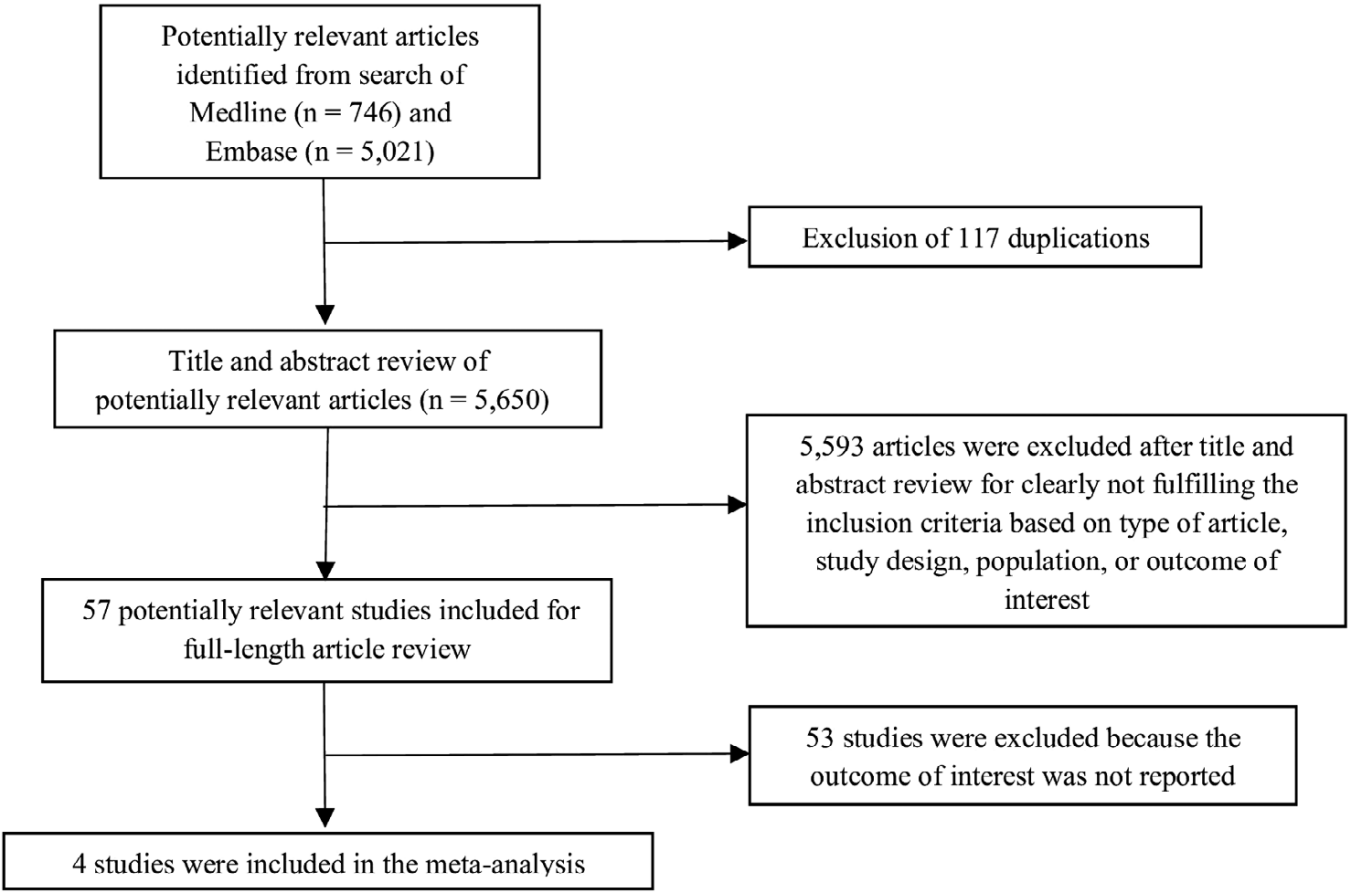
Flow‐chart of the literature review process

The systematic search identified 5767 potentially relevant articles (5021 articles from EMBASE and 746 articles from MEDLINE). After the exclusion of 117 duplicated articles, 5650 articles underwent title and abstract review. A total of 5593 articles were excluded at this stage as they did not fulfill the eligibility criteria based on the type of article, study design, participants, and outcome of interest. A total of 57 articles were retrieved for full‐length article review and 53 articles were excluded at this stage as they did not report the association of interest. Finally, four cohort studies^7^, ^15^, ^16^, ^17^ with 42 677 participants (4651 patients had PVCs) were eligible for the meta‐analysis. The literature retrieval, review, and selection process are shown in Figure 1. The characteristics of the included studies and their quality assessment are described in Table 1.

**TABLE 1:** Baseline characteristics of studies included in the meta‐analysis

	Ofoma et al^15^	Agarwal et al ^16^	Lin et al ^7^	Im et al ^17^
Year of publication	2012	2015	2015	2018
Country of origin	United States	United States	Taiwan	Korea
Study design	Prospective cohort study	Prospective cohort study	Retrospective cohort study	Retrospective cohort study
Study subjects	Cases: Cases were patients with PVCs who were diagnosed based on 2‐minute ECG. Cases were identified from the 1987 to 1989 ARIC study. Comparators: Comparators were the rest of the patients in the study who did not carry a diagnosis of PVCs. The average follow‐up time was 13 years. Patients with a history of stroke, CAD, SAH, and ICH were excluded from the analysis.	Cases: Cases were patients with PVCs who were diagnosed based on routine ECG. Cases were identified from the 2003 to 2007 REGARDS study. Comparators: Comparators were the rest of the patients in the study who did not carry a diagnosis of PVCs. An average (SD) follow‐up time was 6 (2) years. Patients with a history of stroke or TIA and hemorrhagic stroke were excluded from the analysis.	Cases: Cases were patients with PVCs (<720 beats/day) who were diagnosed based on 24‐hour ECG monitoring. Cases were identified from the 2002 to 2004 Taipei Veterans General Hospital database. Comparators: Comparators were the rest of the patients in the database who did not carry a diagnosis of PVCs. The average follow‐up time was 10 ± 1 years. Patients with a history of sustained or non‐sustained VT, PPM, HF, MI, ablation, VHD, and PVCs >720 beats/day were excluded from the analysis.	Cases: Cases were patients with PVCs (>10%) who were diagnosed based on standard ECG or 24‐hour ECG monitoring. Cases were identified from the 2013 to 2015 Kosin University database. Comparators: Comparators were the rest of the patients in the database who did not carry a diagnosis of PVCs. The average follow‐up time was 4 years. Patients with a history of cardiomyopathy, VHD, CHD, hepatic or renal disease, and acute cardiovascular or cerebrovascular event within 3 months, any trauma or surgery within 3 months, hyperthyroidism, uncontrolled HT, malignancy, CTD, IHD, and any acute or chronic inflammatory disease were excluded from the analysis.
Number of subjects	Cases: 793 Comparators: 13700	Cases: 1415 Comparators: 23045	Cases: 1074 (Uniform PVCs), 1166 (Multiform PVCs) Comparators: 1111	Cases: 203 Comparators: 170
Baseline characteristics of subjects	Mean age, year: Cases: 55.97 Comparators: 53.85 Mean BMI, kg/m^2^: Cases: 28.47 Comparators: 27.61 Mean TC, mg/dL: Cases: 211.85 Comparators: 214.69 Female: Case: 50.82% Comparators: 57.06% White: Case: 68.1% Comparators: 73.38% HT: Cases: 44.54% Comparators: 32.80% Diabetes: Cases: 13.98% Comparators: 10.76% Smoking: Cases: 25.73% Comparators: 25.83%	Mean age, year: Cases: 68.3 ± 9.2 Comparators: 64.3 ± 9.3 Mean BMI, kg/m^2^: Cases: 29.2 ± 6.1 Comparators: 29.3 ± 6.2 Mean SBP, mmHg: Cases: 130.1 ± 16.9 Comparators: 126.9 ± 16.4 Female: Cases: 45.4% Comparators: 59% Black: Cases: 41.9% Comparators: 39.9% Education No high school: Cases: 15.5% Comparators: 11.1% High school/no college: Cases: 26.3% Comparators: 25.6% College or professional: Cases: 58.2% Comparators: 63.3% Geographic region Non‐Belt: Cases: 47.2% Comparators: 43.9% Belt: Cases: 33.9% Comparators: 34.8% Buckle: Cases: 19.0% Comparators: 21.3% Previous heart disease: Cases: 25.4% Comparators: 12.2% HT: Cases: 64.7% Comparators: 56.2% DM: Cases: 21.8% Comparators: 19.1% AF: Cases: 9.7% Comparators: 7.8% LVH: Cases: 11.2% Comparators: 9.4% Current smoking: Cases: 13.0% Comparators: 14.0% Use of warfarin: Case: 3.5% Comparators: 2.7% Use of aspirin: Case: 48.1% Comparators: 40.9%	Mean age, year: Cases: 58.7 ± 19.3 (Uniform PVCs), 65.7 ± 16.9 (Multiform PVCs) Comparators: 50.7 ± 20.1 Female: Cases: 44.9% (Uniform PVCs), 31.4% (Multiform PVCs) Comparators: 53.4% DM: Cases: 7.4% (Uniform PVCs), 9.4% (Multiform PVCs) Comparators: 5.6% HT: Cases: 31.2% (Uniform PVCs), 38.0% (Multiform PVCs) Comparators: 21.6% Hyperlipidemia: Cases: 7.4% (Uniform PVCs), 5.8% (Multiform PVCs) Comparators: 5.5% CKD: Cases: 1.4% (Uniform PVCs), 1.4% (Multiform PVCs) Comparators: 0.3% Cirrhosis: Cases: 0.8% (Uniform PVCs),0.8% (Multiform PVCs) Comparators: 0.5% AF: Cases: 6.7% (Uniform PVCs), 6.8% (Multiform PVCs) Comparators: 6.7% CLD: Cases: 2.6% (Uniform PVCs), 2.8% (Multiform PVCs) Comparators: 2.0% Use of AAD: Cases: 0.2% (Uniform PVCs), 0.5% (Multiform PVCs) Comparators: 0.5% Use of anti‐HT: Cases: 16.4% (Uniform PVCs), 20.8% (Multiform PVCs) Comparators: 12.2% Use of statin: Cases: 7.2% (Uniform PVCs), 5.7% (Multiform PVCs) Comparators: 5.1%	Mean age, year: Cases: 61.0 ± 15.2 Comparators: 57.6 ± 16.4 Female: Cases: 56.7% Comparators: 52.7% DM: Cases: 17.5% Comparators: 20.0% HT: Cases: 28.4% Comparators: 30.6% CAD: Cases: 11.3% Comparators: 12.4% Medication Amiodarone: Cases: 7.7% Comparators: 0% Propafenone: Cases: 1.0% Comparators: 0% Digoxin: Cases: 5.2% Comparators: 4.8% Beta‐blocker: Cases: 53.6% Comparators: 8.2% CCB: Cases: 21.6% Comparators: 20% ARB & ACEI: Cases: 17.0% Comparators: 16.4% Statin: Cases: 39.4% Comparators: 37.6% Aspirin: Cases: 24.6% Comparators: 25.3% Clopidogrel: Cases: 13.4% Comparators: 12.0% VKA: Cases: 9.8% Comparators: 11.2% Laboratory findings Mean WBC, 10^3^/μL: Cases: 7.4 ± 2.7 Comparators: 7.8 ± 3.0 Mean Cr, mg/dL: Cases: 1.2 ± 0.6 Comparators: 1.3 ± 0.9 Mean TSH, mg/dL: Cases: 2.7 ± 1.6 Comparators: 2.8 ± 1.7 Mean FT4: Cases: 1.2 ± 0.4 Comparators: 1.2 ± 0.5 Mean Pro‐BNP: Cases: 1469.5 ± 876.4 Comparators: 1281.1 ± 231.5 Echo‐parameters Mean LVEF, %: Cases: 62.9 ± 13.5 Comparators: 59.1 ± 11.9 Mean LVIDs, mm: Cases: 34.5 ± 7.9 Comparators: 31.2 ± 9.4 Mean LVIDd, mm: Cases: 50.6 ± 6.6 Comparators: 46.8 ± 8.0 Mean IVSD, mm: Cases: 10.8 ± 2.7 Comparators: 12.1 ± 3.7 Mean LVPWD, mm: Cases: 9.9 ± 2.2 Comparators: 10.4 ± 2.2 Mean LAVi, mL/m^2^: Cases: 24.8 ± 12.1 Comparators: 22.1 ± 17.9 Mean E velocity, cm/s: Cases: 0.8 ± 0.3 Comparators: 0.7 ± 0.2 Mean A velocity, cm/s: Cases: 0.7 ± 0.2 Comparators: 0.7 ± 0.2 Mean E': Cases: 0.1 ± 0.04 Comparators: 0.1 ± 0.03 Mean E/E': Cases: 11.0 ± 7.0 Comparators: 11.4 ± 6.6
Diagnosis of PVCs	PVCs were detected by 2‐minute ECG.	PVCs were detected by routine ECG.	PVCs were detected by 24‐hour Holter monitoring.	PVCs were detected by standard ECG or 24‐hour Holter monitoring.
Diagnosis of stroke	The diagnosis of ischemic stroke was supported by annual telephone follow‐up and a community surveillance system. Stroke was diagnosed by agreement of computer and reviewer classification. Disagreements were adjudicated by a second physician‐reviewer.	The diagnosis of ischemic stroke was supported by telephone follow‐up every 6 months. Stroke events were defined following the WHO definition. The stroke was diagnosed and confirmed by an adjudication committee.	Ischemic stroke was diagnosed by physicians and also confirmed by brain imaging.	Ischemic stroke was determined by a neurologist if the patient felt painless, weakness, sudden numbness or dead feeling on one side of the body, sudden painless loss of vision, and sudden loss of ability to understand what people were saying and also confirmed by brain MRI in all patients.
Confounder adjusted in the multivariate analysis	Age, race, gender, BMI, HT, diabetes, smoking, TC levels	Age, sex, race, geographic region, education level, previous heart disease, SBP, use of antihypertensive medication, LVH, AF, DM, current smoking, use of warfarin and aspirin	Age, sex, HT, DM, CKD, use of hypertensive medication	Age, AF, HT, LVH, E/E', MR grade, NT‐proBNP
Newcastle‐Ottawa score	Selection: 4 stars Comparability: 2 stars Outcome: 3 stars	Selection: 4 stars Comparability: 2 stars Outcome: 3 stars	Selection: 3 stars Comparability: 1 star Outcome: 3 stars	Selection: 4 stars Comparability: 2 stars Outcome: 3 stars

Abbreviations: A, peak velocity of the late filling wave due to atrial contraction; AAD, antiarrhythmic drug; ACEI, angiotensin‐converting enzyme inhibitor; AF, atrial fibrillation; ARB, angiotensin II receptor blocker; ARIC, atherosclerosis risk of communities; BMI, body mass index; CAD, coronary artery disease; CCB, calcium channel blocker; CHD, congenital heart disease; CKD, chronic kidney disease; CLD, chronic lung disease; CTD, connective tissue disease; DM, diabetes mellitus; ECG, electrocardiogram; E, the peak mitral flow velocity of the early rapid filling wave; E', early diastolic mitral annulus velocity; fT4, free thyroxine 4; HF, heart failure; HT, hypertension; ICH, intracerebral hemorrhage; IHD, ischemic heart disease; IVSD, interventricular septal diameter; LAVi, left atrial volume index; LVEF, left ventricular ejection fraction; LVH, left ventricular hypertrophy; LVIDd, left ventricular diastolic diameter; LVIDs, left ventricular systolic diameter; LVPWD, left ventricular posterior wall dimension; MI, myocardial infarction; MR, mitral regurgitation, MRI, magnetic resonance imaging; NT‐proBNP, N‐terminal pro‐B type natriuretic peptide; PPM, permanent pacemaker; pro‐BNP, pro‐B type natriuretic peptide; PVCs, premature ventricular contractions; REGARDS, reasons for geographic and racial differences in stroke; SAH, subarachnoid hemorrhage; SBP, systolic blood pressure; SD, standard deviation; TC, total cholesterol; TIA, transient ischemic attack; VHD, valvular heart disease; VKA, vitamin K antagonist; VT, ventricular tachycardia; WBC, white blood cell count; WHO, world health organization.

### 3.1 Risk of ischemic stroke among patients with PVCs

**FIGURE 2:**
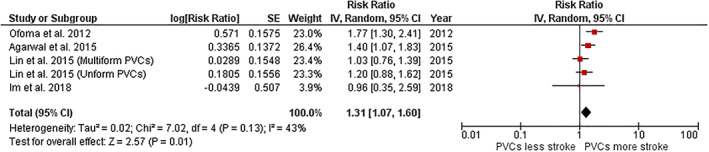
Forest plot of the meta‐analysis

A total of four studies reported on the risk of ischemic stroke among patients with PVCs.^7^, ^15^, ^16^, ^17^ The pooled analysis found a significantly increased risk of ischemic stroke among patients with PVCs compared to individuals without PVCs with the pooled RR of 1.31 (95% CI, 1.07–1.60). The between‐study heterogeneity was low with an I^2^ of 43%. Figure 2 demonstrates the forest plot of this meta‐analysis.

### 3.2 Sensitivity analysis

To further explore the high between‐study heterogeneity, a sensitivity analysis was conducted by excluding the study by Lin et al^7^ from the complete analysis as it was the only one study that had low burdens of PVCs (PVCs <720 beats per day), and it was the study with the lowest Newcastle‐Ottawa score. This sensitivity analysis did not significantly alter the pooled result (pooled RR 1.52; 95% CI, 1.23–1.87) but dramatically decreased I^2^ to an insignificant level (6%).

### 3.3 Evaluation for publication bias

Evaluation for publication bias using the funnel plot was not performed due to the limited number of included studies which would preclude a meaningful interpretation of the plot.

## 4 DISCUSSION

The current study is the first systematic review and meta‐analysis that summarized data from all available studies on the risk of ischemic stroke among patients with PVCs and found about 30% excess risk compared to individuals without PVCs. There are possible mechanisms to explain this observed increased risk.

The first possible explanation is that PVCs are associated with the development of ventricular cardiomyopathy.^21^ PVCs can lead to reduced expression of calcium channel, voltage‐dependent, L‐type, alpha 1C subunit (Cav1.2), density of L‐type calcium (I_CaL_) current, and relocated Cav1.2 away from t‐tubules in the ventricular myocardium which would subsequently lead to abnormal calcium‐induced calcium release from the sarcoplasmic reticulum.^22^ In the event of long‐term frequent PVCs, this process may eventually lead to cardiac remodelings such as apoptosis of myocardial cells and fibrosis, resulting in permanent structural changes of the ventricle.^16^, ^23^, ^24^ One of the frequent structural changes seen in patients with ventricular cardiomyopathy is ventricular dilatation, which is known to provoke the stasis of blood flow, increased risk of thrombus formation, and embolism.^25^, ^26^ Due to the unique hemodynamic changes occurring in patients with PVCs, irregular ventricular rhythm as well as variable coupling intervals can also cause stasis of blood flow and promote thrombus formation in the left atrial appendage.^27^ Moreover, the ventricular contraction from PVCs could not provide an effective cardiac output as a result of mechanical ventricular dyssynchrony. This leads to a transient decline in arterial blood pressure which could deteriorate the regionally and globally cerebral circulation.^28^, ^29^, ^30^

The second explanation is the increased risk of subsequent AF, the prime predisposing of thromboembolic events.^7^, ^17^, ^26^, ^31^ Frequent PVCs may not only lead to ventricular cardiomyopathy but also atrial cardiomyopathy as well. This could be from an indirect consequence of ventricular dysfunction as it could increase atrial pressure and volume overload. This ultimately results in myocardial stretching and development of fibrosis in the atrial walls.^32^ Therefore, atrial fibrosis resulting from atrial remodeling can lead to conduction abnormalities to sustain AF.^33^ However, this hypothesis is somewhat speculative as most studies did not provide any information on the timing of the onset of AF. Thus, it could not draw the exact association between PVCs, AF, and ischemic stroke. On the other hand, most studies just reported the incidence of new‐onset AF at the end of follow‐up.^7^, ^17^, ^26^, ^31^

Thirdly, the apparent association between PVCs and ischemic stroke might be explained by the higher rate of cardiovascular disease in patients with PVCs. Although PVCs have been historically considered benign,^2^ it is known to be associated with structural heart disease and increased risk of sudden cardiac death, suggesting that PVCs could be a consequence of conductive tissue injury from associated cardiovascular diseases.^34^, ^35^ Thus, it is also possible that the presence of PVCs simply reflects a higher burden of cardiovascular disease, which contributes to the increased risk of ischemic stroke.^26^ However, patients who had PVCs may need more intensive monitoring and treatment, even if it is not known with the certainty whether ventricular and/or atrial cardiomyopathy from PVCs was directly responsible for the ischemic stroke or the presence of PVCs was just a marker of a high burden of atherosclerosis. Further prospective studies are still needed to determine whether a true causal mechanism exists between PVCs and ischemic stroke.

Although the included studies were of high quality and the literature review process was thorough, we acknowledge that our study has some limitations and the results should be interpreted with caution. Firstly, this meta‐analysis had some between‐study heterogeneity in the ischemic stroke analysis. The difference in PVCs burden across the included studies was the most likely explanation for the heterogeneity as demonstrated by the sensitivity analysis. Secondly, most of the included studies used the different measurement methods to detect and diagnose PVCs: 2‐minute ECG was used in 1 study,^15^ routine ECG in 1 study,^16^ and 24‐hour Holter monitoring in 2 studies.^7^, ^17^ Therefore, the accuracy of the diagnoses from those studies was relatively limited. Lastly, evaluation for publication could not be conducted because of the limited number of included studies. Therefore, publication bias in favor of studies that report positive results may have been present.

## 5 CONCLUSION

The current study found that the presence of PVCs is associated with a higher risk of ischemic stroke. Whether this association is causal and how it should be addressed in clinical practice require further investigations.

## CONFLICT OF INTEREST

All authors declare no personal or professional conflicts of interest, and no financial support from the companies that produce and/or distribute the drugs, devices, or materials described in this report.

## AUTHOR CONTRIBUTIONS

All authors designed the study. Pongprueth Rujirachun, Phuuwadith Wattanachayakul, and Prawut Phichitnitikorn collected the data and drafted the manuscript. Pongprueth Rujirachun performed the statistical analysis. Pongprueth Rujirachun, Nipith Charoenngam, Jakrin Kewcharoen, and Arjbordin Winijkul made critical revisions. Pongprueth Rujirachun, Nipith Charoenngam and Jakrin Kewcharoen revised the final manuscript. All authors read and approved the final manuscript.

## Supporting information


**Appendix** **S1**: Supporting Information

**Appendix** **S2**: Supporting Information

## Data Availability

The data that supports the findings of this study is available on request from the corresponding author.
