# Supplementary material for: Association of premature ventricular complexes and risk of ischemic stroke: A systematic review and meta‐analysis
Source: Clin Cardiol. 2020 Dec 16;44(2):151–9. doi: 10.1002/clc.23531 (PMC7852158; doi:10.1002/clc.23531)
Supplement: Supplementary file 1 — Appendix S1: Supporting Information [file CLC-44-151-s001.docx]

**Supplementary data 1**

Search strategy

**EMBASE**

1. 'pvc'/exp OR 'pvc'
2. 'heart ventricular extrasystole'/exp OR 'heart ventricular extrasystole'
3. 'heart ventricular arrhythmia'/exp OR 'heart ventricular arrhythmia'
4. 'heart ventricular bigeminy'/exp OR 'heart ventricular bigeminy'
5. 'ventricular ectopy'/exp OR 'ventricular ectopy'
6. 'premature ventricular complex'/exp OR 'premature ventricular complex'
7. ('premature'/exp OR 'premature') AND ventricular AND ('contraction'/exp OR 'contraction')
8. 'cerebrovascular accident'/exp OR 'cerebrovascular accident'
9. 'cerebrovascular disease'/exp OR 'cerebrovascular disease'
10. 'stroke patient'/exp OR 'stroke patient'
11. 'stroke unit'/exp OR 'stroke unit'
12. 'brain ischemia'/exp OR 'brain ischemia'
13. 'lacunar infarct'/exp OR 'lacunar infarct'
14. 'lacunar stroke'/exp OR 'lacunar stroke'
15. 'brain infarction'/exp OR 'brain infarction'
16. 'transient ischemic attack'/exp OR 'transient ischemic attack'
17. #1 OR #2 OR #3 OR #4 OR #5 OR #6 OR #7
18. #8 OR #9 OR #10 OR #11 OR #12 OR #13 OR #14 OR #15 OR #16
19. #17 AND #18

**Medline**

1. exp Ventricular Premature Complexes/ or ventricular ectopy.mp.
2. exp Ventricular Premature Complexes / or ventricular premature contraction.mp.
3. exp Ventricular Premature Complexes / or premature ventricular complex.mp.
4. exp Ventricular Premature Complexes / or ventricular extrasystole.mp.
5. PVC.mp.
6. Ventricular arrhythmia.mp.
7. Cerebrovascular damage.mp.
8. Cerebrovascular accident.mp.
9. exp Stroke/ or stroke.mp.
10. exp Stroke, Lacunar/ or lacunar stroke.mp.
11. exp Cerebrovascular Disorders/ or cerebrovascular event.mp.
12. exp Brain Ischemia/ or ischemic stroke.mp.
13. exp Ischemic Attack, Transient/ or TIA.mp.
14. exp “Intracranial Embolism and Thrombosis”/ or exp Embolism/ or embolism.mp.
15. or/1-6
16. or/7-14
17. 15 and 16
